# Update on Genetic Chorea

**DOI:** 10.1007/s11910-026-01502-5

**Published:** 2026-07-04

**Authors:** Miriam Ostrozovicova, Matej Skorvanek

**Affiliations:** 1 Department of Neurology, University of Pavol Jozef Safarik, Trieda SNP 1, Kosice, 04011 Slovakia; 2 Department of Neurology, University Hospital L. Pasteur, Kosice, Slovakia; 3 Department of Clinical Neurosciences, University of Pavol Jozef Safarik, Kosice, Slovakia; 4 Neuromuscular Disease Department, UCL Queen Square Institute of Neurology, University College London, London, WC1N 3BG UK

**Keywords:** Chorea, Genetics, Huntington’s disease, Somatic instability, Benign hereditary chorea, Huntington’s disease look-alikes

## Abstract

**Purpose of Review:**

Chorea is a symptom of numerous pathophysiologically and clinically heterogeneous genetic conditions. A number of developments have been made in this field over the last years linked to improved genomic testing, large cohort collaborations and improved understanding of the molecular mechanisms. This review aims to provide an update on the new genetic conditions and phenotypes linked to chorea disorders, their modification factors and pathophysiological background.

**Recent Findings:**

Several novel genetic conditions have been linked to chorea over the last 3 years, including mutations in *FTH1*, *NAA60*, *ACBD6* or *TOR1AIP2*. Also, novel phenotypes have been established and linked to chorea, such as Adult-onset Neurodegeneration in Nucleotide Excision Repair Disorder (NERD-ND). Major advances have been made in understanding of the pathophysiological role of somatic instability in HD. Striatal pallidal neurons (SPNs) with 150–500 + CAG repeats seem to lose positive and then negative features of neuronal identity, de-repress senescence/apoptosis genes, ultimately leading to cell death.

**Summary:**

Improved recognition of the genetic background of chorea leads to more effective diagnostic processes, better prognostication and improved personalized treatment. The findings on somatic instability in HD suggest that neurodegeneration in HD is an asynchronous DNA process for >95% of a neuron's life, with majority of neurons in all disease stages having a HTT gene which is not biologically harmful. This has potential major therapeutic implications not only in HD but also in other neurological repeat expansion disorders.

## Introduction

Chorea is a hyperkinetic movement disorder, characterized by brief, abrupt, irregular, unpredictable, and non-stereotyped involuntary movements. It represents a major phenotypic manifestation of many disorders with a continuously expanding spectrum of associated genetic and acquired etiologies. The differential diagnosis of rare movement disorders, including chorea, is broad and may be challenging. Nevertheless, as with other movement disorders like tremor [1] or dystonia [2], the diagnostic algorithm should generally follow 2 steps - (a) clinical characterization of the disease, including detailed phenotypic and syndromic classification and (b) additional investigations, incl. imaging and laboratory analyses that support etiological classification of the disease. Age at onset constitutes one of the most critical factors in the differential diagnosis of chorea. Huntington’s disease (HD) should be the most important disorder to consider in adult-onset cases, after drug-induced chorea has been excluded, followed by other genetic or acquired etiologies. In contrast, childhood-onset chorea is most commonly associated with autoimmune conditions, although in younger children a number of genetic conditions need to be considered as well in the first line [3]. In recent years, substantial advancement have been made in the understanding of genetic chorea syndromes, including discovery of new genes and phenotypes, the recognition of genetic modifiers, and improved insights into underlying pathophysiological mechanisms. This review aims to outline the relevant updates in the field of genetic chorea.

### Huntington’s Disease

**Fig. 1:**
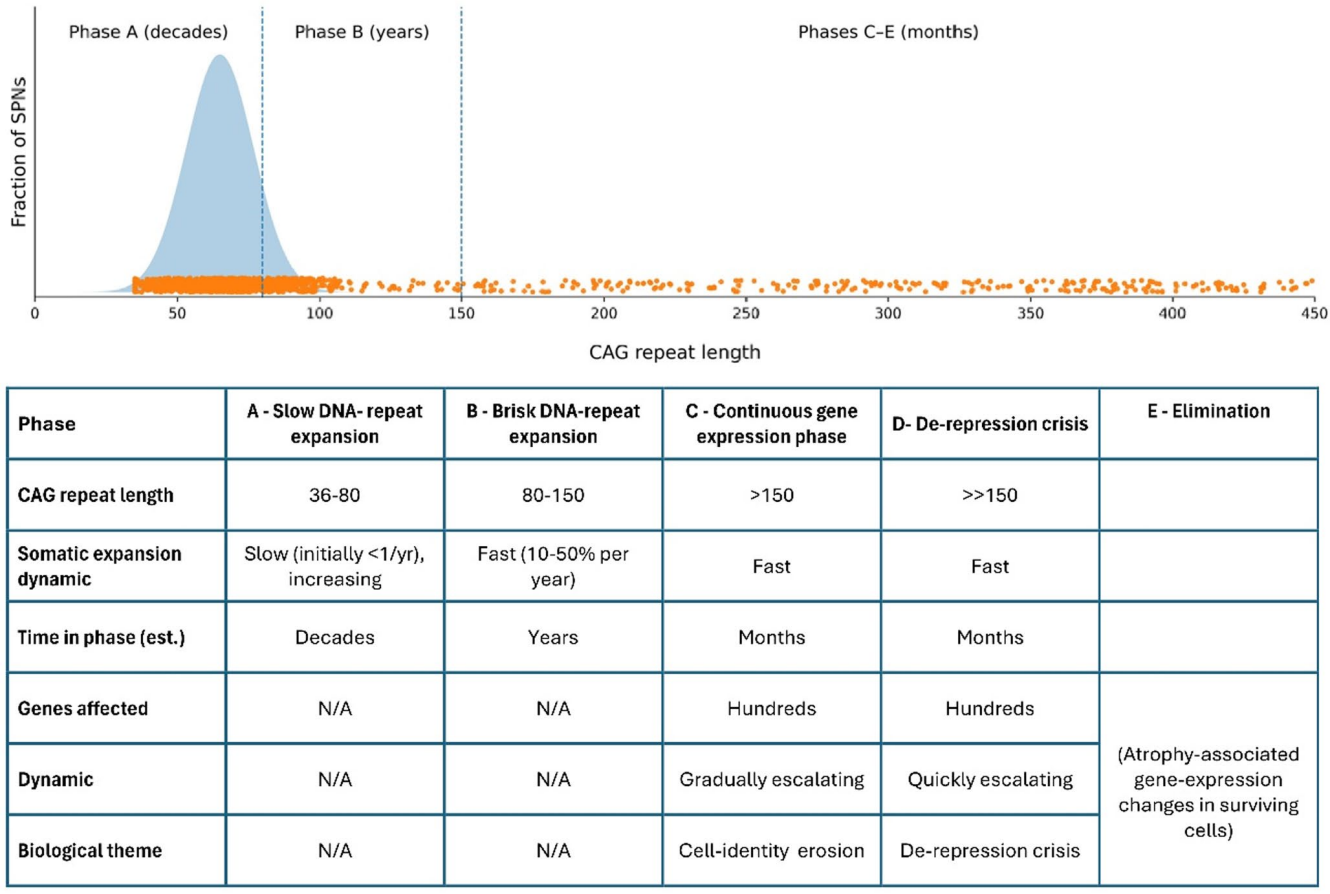
ELongATE: A model for SPN pathology in HD. Individual neurons pass asynchronously through five key pathological phases, spending >95% of their lives in a long period of DNA-repeat expansion (a ticking DNA clock, phases A and B) with a biologically harmless (but unstable) HTT gene. Individual neurons asynchronously exit phase A and proceed through the subsequent, faster phases (modified from Handsaker et al. 2025)

HD is an autosomal dominant disease caused by expansion of (CAG)n triplet repeats within exon 1 of the *huntingtin (HTT)* gene [4]. In its classical form it is characterised by a combination of chorea, cognitive problems and psychiatric disturbances. The disease follows a progressive course, typically beginning with mild behavioral and cognitive changes, followed by chorea which later progresses into dystonia and parkinsonism [5]. Typical gait impairment is often caused by a combination of chorea, dystonia, parkinsonism and frontal apraxia of gait [6]. In addition, patients often present with oculomotor abnormalities including oculomotor apraxia, impaired fixation, slow and hypometric saccades in both the vertical and horizontal planes [7]. Juvenile-onset HD may present with atypical phenotypes dominated by parkinsonism and dystonia rather than chorea, combined with myoclonus, ataxia, tics, seizures and developmental delay [8]. In addition to the caudate atrophy typically observed in the classic adult-onset form. CAG repeat lengths of 36 or more are considered pathogenic, with longer expansions generally associated with earlier disease onset albeit with substantial inter-individual variability (36–55 in 98% of affected individuals, 40–49 in 90%) [9]. Expansions between 36 and 39 CAG units confer reduced penetrance [10], suggesting that some individuals carrying these reduced penetrance alleles might have HD with disease onset beyond the normal lifespan. Multiple genetic modifiers of HD have been identified. Results from the Genetic modifiers of Huntington’s Disease (GeM-HD) consortium show that timing of HD onset correlates with the length of uninterrupted CAG repeat sequence rather than with the polyglutamine length, which may be interrupted by glutamine-coding CAA interruptions [11]. These findings suggest that the special onset-determining property of the uninterrupted CAG repeat is a propensity for length instability that leads to its somatic expansion. In addition, age at onset may be modified by common genetic variation at several genes, many of them having DNA maintenance function, including *MSH3*,* FAN1*,* MLH1*,* LIG1*,* PMS1 and PMS2* [11, 12], which affect DNA-repeat stability [13–15]. In a recent landmark report, Handsaker et al. [16] provided a novel major development in understanding of HD pathophysiology based on single-cell method for measuring the somatic repeat’s length alongside genome-wide RNA expression. Based on their findings striatal projection neurons (SPN) are specifically vulnerable to somatic repeat expansions, with specific biological effects measured at different levels of repeat expansion (Fig. 1). In their findings somatic expansion from 40 to 150 CAGs had no apparent cell autonomous effect, but SPNs with 150–500 + CAGs lost positive and then negative features of neuronal identity, de-repressed senescence/apoptosis genes, and were lost [16]. These results suggest that neurodegeneration in HD is an asynchronous DNA process for > 95% of a neuron’s life, with most of the still-living SPNs having a *HTT* gene that is not yet biologically harmful. Almost all therapies in advanced clinical development for HD so far have been focused on lowering HTT expression with unsuccessful results. Nevertheless, based on results of Handsaker et al. [16], only a small minority of SPNs may have toxic HTT protein at the time of the therapeutic trial and the expected lifetime of SPNs that reach the phases of cell/biological toxicity are very short, potentially limiting the overall benefit of these HTT lowering strategies. Therefore, a shift towards strategies aimed at slowing somatic expansion, supported by some preclinical cell and animal models [13, 17, 18], is of great interest.

### Huntington’s Disease Mimics

**Fig. 2:**
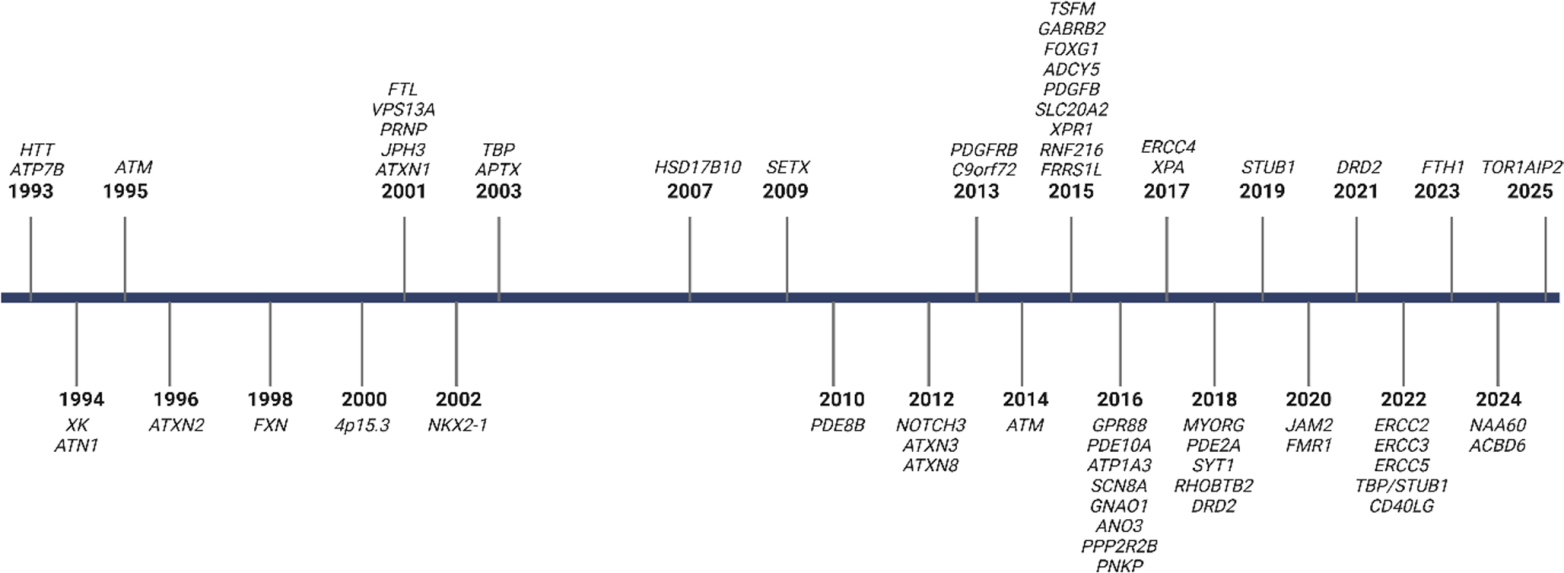
Timeline of genes implicated in chorea: year of the first published genotype-phenotype correlation study

Approximately 1% of patients presenting with the typical HD phenotype, characterized by chorea with cognitive impairment and psychiatric disturbances, do not harbour a pathogenic *HTT* CAG expansion [19]. These conditions are known as HD phenocopies, HD look-alikes, or HD mimics. Prior to the advent of modern DNA sequencing technologies, the diagnosis of HD relied exclusively on clinical evaluation and neuropathological findings. Nowadays, genotype-phenotype studies have led to a rapid and substantial expansion in the list of genetic conditions associated with chorea (Fig. 2, and Table 1). Although many of these disorders have long been well recognized, several substantial new insights into their underlying mechanisms, as well as the identification of previously unappreciated phenotypic features that have refined and expanded their clinical characterization, have emerged in recent years.

**Table 1:** Summary of genetic causes of chorea syndromes

Condition	Gene	Inheritance	Age of onset	Additional clinical features	Neuroimaging
*Chorea caused by Repeat Expansions*					
HD	*HTT*	AD	juvenile to late adulthood	oculomotor dysfunction, motor impersistence, tics/ vocalisations, parkinsonism, dystonia, myoclonus, psychiatric symptoms, cognitive decline	caudate nucleus head atrophy
*Huntington’s Disease-like Syndromes (HDL)*					
HDL1	*PRNP*	AD	adulthood	psychiatric symptoms, dementia, myoclonus, ataxia, parkinsonism, seizures	cortical and caudate nucleus atrophy
HDL2	*JPH3*	AD	adulthood	parkinsonism, cognitive decline, psychiatric symptoms	cortical ribboning, basal ganglia hyperintensity on DWI, generalised atrophy
HDL3	linked to 4p15	AR	juvenile onset	chorea, dystonia, ataxia, spasticity, seizures, mutism	frontal cortical atrophy and bilateral caudate atrophy
HDL4 / SCA17	*TBP1*	AD	juvenile to adulthood	ataxia, parkinsonism, cognitive decline,	cerebellar and frontal lobes atrophy
* C9orf72* repeat expansions	*C9orf72*	AD	juvenile to adulthood	FTD, ALS, ataxia, dystonia, tremor, myoclonus, parkinsonism, prominent cognitive decline and psychiatric symptoms, hallucinations	frontal and temporal atrophy, thalamic atrophy,
*Chorea in Cerebellar Ataxia-related Genes*					
SCA1	*ATXN1*	AD	adulthood	ataxia, pyramidal signs, slow saccades, bulbar symptoms, cognitive decline	cerebellar and brainstem atrophy
SCA2	*ATXN2*	AD	early adulthood	ataxia, slow saccades, peripheral neuropathy, parkinsonism	cerebellar and pontine atrophy
SCA3	*ATXN3*	AD	adulthood	ataxia, oculomotor apraxia, pyramidal signs, parkinsonism, peripheral neuropathy	cerebellar and brainstem atrophy
SCA8	*ATXN8/ ATXN8OS*	AD	adulthood	ataxia, impaired smooth pursuits, saccadic intrusions, mild cognitive impairment, psychiatric features	mild cerebellar atrophy
SCA12	*PPP2R2B*	AD	adulthood	ataxia, action/postural tremor	cerebellar atrophy, mild cortical atrophy
DRPLA	*ATN1*	AD	juvenile to adulthood	psychiatric symptoms, seizures, myoclonus, ataxia, cognitive decline	cerebellar and brainstem atrophy
FXTAS	*FMR1*	XR	adulthood	ataxia, parkinsonism, intention tremor, executive dysfunction, cognitive decline, peripheral neuropathy	T2-weighted hyperintensities of the middle cerebellar peduncles
Ataxia- telangiectasia	*ATM*	AR	juvenile to adulthood	ataxia, gait apraxia, chorea, myoclonus, dystonia, oculocutaneous telangiectasias, oculomotor apraxia, axonal sensorimotor neuropathy, immunodeficiency, cancer susceptibility *Lab tests*: *↑* AFP	cerebellar atrophy
Ataxia with oculomotor apraxia type 1, 2, and 4	*APTX* *SETX* *PNKP*	AR	juvenile to adulthood	sensorimotor neuropathy, oculomotor apraxia, ataxia *Lab tests*: *↓* albumin (type 1) *↑* cholesterol (type 1,4) *↑* AFP (type 2,4)	cerebellar atrophy
Friedreich ataxia	*FXN*	AR	juvenile to late adulthood	muscle weakness, scoliosis, sensory neuropathy, cardiomyopathy, pes cavus	brainstem, cerebellar and spinal cord atrophy
* RNF216*-mediated neurodegeneration (Gordon-Holmes syndrome)	*RNF216*	AR	adulthood	hypogonadotropic hypogonadism, ataxia, behavioural problems, and severe dementia, absent inferior incisors	white matter lesions, cerebellar atrophy
Adult-Onset Neurodegene-ration in Nucleotide Excision Repair Disorders	*ERCC2 ERCC3 ERCC4 ERCC5 XPA*	AR	early to late adulthood	ataxia, dementia, neuropathy, hearing impairment, photosensitivity, skin freckling, skin neoplasms	global atrophy
*Chorea in Primary Immunodeficiency-related Genes*					
X-linked IgM immunodefi-ciency	*CD40LG*	XLR	adulthood	cognitive decline, psychiatric disturbances, ataxia	global atrophy
*Chorea caused by SNV or CNV*					
*Neuroacanthocytosis Syndromes*					
Chorea- acanthocytosis	*VPS13A*	AR	early adulthood	feeding dystonia with self-mutilation, sudden head drops, trunk spasms, abnormal vocalisations/ tics, ataxia, psychiatric features, seizures, myopathy, peripheral neuropathy *Lab tests*: ↑ serum CK acanthocytosis	Atrophy of the caudate nucleus head
McLeod syndrome	*XK*	XR	adulthood	ataxia, cognitive impairment, psychiatric symptoms, cardiomyopathy, neuromuscular involvement, peripheral neuropathy *Lab tests*: ↑ serum CK acanthocytosis	cortical, basal ganglia and cerebellar atrophy
*Inborn errors of copper metabolism*					
Wilson‘s disease	*ATP7B*	AR	juvenile to adulthood	oculomotor dysfunction, abnormal vocalisations/ tics, parkinsonism, dystonia, psychiatric symptoms Lab tests: ↓ levels of serum copper ↓ serum ceruloplasmin ↑ hepatic transaminase levels ↑ 24-hour urine copper excretion aminoaciduria hemolytic anemia	T2 hyperintensities in the basal ganglia, thalamus, brainstem (“giant panda sign”), cerebral and cerebellar atrophy
*Basal Ganglia Calcification syndrome*					
Primary Brain Calcification	*SLC20A2* *XPR1* *PDGFB PDGFRB*	AD	early to late adulthood	parkinsonism, psychosis with manic features	calcifications in basal ganglia, cerebellar dentate nuclei and subcortical white matter
	*MYORG JAM2 NAA60*	AR			
*Neurodegeneration with brain iron accumulation*					
Neuroferritino- pathy	*FTL1*, *FTH1*	AD	juvenile to late adulthood	action-specific facial dystonia, parkinsonism, cognitive behavioural issues *Lab tests*: *↓* Ferritin plasma levels	brain iron accumulation in basal ganglia, cortical pencil lining
*Benign hereditary chorea*					
* NKX1-1*-related neurodevelop-mental disorder	*NKX2-1*	AD/De novo	juvenile onset	dystonia, myoclonus, ataxia, axial hypotonia; pulmonary disease or congenital hypothyroidism	nonspecific, rarely mild cerebral and cerebellar atrophy
* ADCY5*-related Movement Disorder	*ADCY5*	AD/AR	juvenile onset	dystonia, myoclonus, ataxia, axial hypotonia, facial myokymia, diurnal and nocturnal exacerbations	nonspecific, rarely mild cerebral and cerebellar atrophy
*Chorea in Epileptic Encephalopathy-related Genes*					
Early infantile epileptic encephalopa-thy type 17 (Ohtahara syndrome)	*GNAO1*	De novo	infancy to adulthood	developmental delay, seizures	normal, rarely delayed myelination or mild cerebral and cerebellar atrophy
Congenital Rett disease	*FOXG1*	De novo	infancy to early childhood	severe intellectual disability, microcephaly	hypoplasia or agenesis of corpus callosum, frontal or frontotemporal underdevelopment mild cerebellar hypoplasia, delayed myelination
Severe motor delay and intellectual disability	*SYT1*	De novo	Infancy	Severe delayed motor development without seizures	normal or mild cerebral and cerebellar atrophy
Early infantile epileptic encephalopa-thy type 13 / BFIS	*SCN8A*	AD / De novo	Infancy to childhood	paroxysmal dystonia, focal EEG abnormalities	normal or mild cerebral and cerebellar atrophy
* GABRB2*- Associated Neurodevelop-mental Disorders	*GABRB2*	De novo	Infancy to childhood	early myoclonic encephalopathy, developmental delay, microcephaly, dystonia, ataxia	normal or mild cerebral atrophy
* FRRS1L-*mediated chorea	*FRRS1L*	AR	juvenile onset	epileptic encephalopathy, continuous spikes and waves during sleep	normal or white matter lesions, cortical and cerebellar atrophy
* HSD10* mitochondrial disease	*HSD17-* *B10*	XLD	juvenile onset	refractory epilepsy, learning disability	normal or frontotemporal atrophy, basal ganglia abnormality, periventricular white matter disease
*Chorea in Dystonia-related Genes*					
* TOR1AIP2-* related dystonia	*TOR1-* *AIP2*	AR	juvenile	dystonia, hemibalism, hemichorea	normal
Rapid-onset Dystonia- Parkinsonism	*ATP1A3*	AD	juvenile to early adulthood	sudden onset of dystonia, parkinsonism triggered by stress	normal
* DRD2-*Child-Onset Chorea	*DRD2*	AD	juvenile to early adulthood	cervical dystonia, vertical eye movement apraxia, hypotonia, developmental delay	normal
DYT24 dystonia	*ANO3*	AD	juvenile to early adulthood	craniocervical dystonia, tremor, myoclonus	normal
*Chorea in Neurodevelopmental Disorders-Related Genes*					
* TSFM-*Early-Onset Complex Chorea	*TSFM*	AR	juvenile to early adulthood	slowly progressive optic atrophy, cardiomyopathy, ataxia, myoclonus, dystonia	normal or lesions in the basal ganglia
Intellectual developmental disorder with paroxysmal dyskinesia or seizures	*PDE2A*	AR	juvenile	developmental delay, hypotonia, intellectual disability	normal or subtle atrophy
Autosomal- dominant striatal degeneration	*PDE8B*	AD	juvenile	dystonia, ataxia, developmental delay	striatal changes
Striatal degeneration, autosomal dominant 2	*PDE10A*	De novo / AD / AR	infancy to childhood	peculiar foot deformities, social phobia, adult-onset parkinsonism, hypotonia, microcephaly, seizures, dystonia, dysarthria, developmental delay	normal or symmetrical bilateral striatal lesions
Chorea, childhood-onset, with psychomotor retardation	*GPR88*	AR	infancy to childhood	developmental delay, intellectual disability,	normal
Neurodevelopmental disorder with progressive movement abnormalities	*ACBD6*	AR	infancy	developmental delay, intellectual disability, behavioural abnormalities, dystonia, spasticity, cerebellar ataxia, microcephaly, dysmorphic features	callosum abnormalities, hypoplasia or agenesis of the anterior commissure, short midbrain, small cerebellar vermis, enlarged ventricles, incomplete hippocampal inversion
Developmental and epileptic encephalopa-thy 64	*RHOBTB2*	AD	infancy	neurodevelopmental delay, intellectual disability, epileptic encephalopathy, dysmorphic features	delayed myelination, thin corpus callosum, enlarged ventricles, cortical atrophy, cerebellar hypoplasia
CADASIL	*NOTCH3*	AD	adulthood	migraine, strokes, dementia	leukoencephalopathy, cerebral microvascular ischemic change affecting the lentiform nucleus, thalamus and brainstem

*AD *Autosomal dominant, *AR *Autosomal recessive, *XLR *X-linked recessive, *XLD *X-linked dominant, *HD *Huntington’s disease, *HDL *Huntington’s disease-like, *SCA *Spinocerebellar Ataxia, *DRPLA *Dentatorubral–pallidoluysian atrophy. It is a rare, autosomal dominant neurodegenerative disorder, *FXTAS *Fragile X–associated tremor/ataxia syndrome, *SNV *Single Nucleotide Variant, *CNV *Copy Number Variant, BFIS Benign familial infantile seizures, *CADASIL *Cerebral Autosomal Dominant Arteriopathy with Subcortical Infarcts and Leukoencephalopathy

### Huntington’s Disease-Like Syndromes (HDL)

Although HDL syndromes are rare, they are important to consider in every patient with a negative result from HD genetic testing, if acquired causes are excluded. Recent observations emphasize expanding phenotypic spectrum and disease mechanism. In contrast with HD, in HDL1 linked to the prion *PRPN* gene ataxia and psychiatric symptoms are often predominant [20]. HDL2, associated with the *JPH3* gene CAG/CTG repeat expansion, is nearly indistinguishable from HD, although atypical cases have been recently reported presenting with parkinsonism-dystonia in combination with cognitive and behavioral impairment [21], or with peculiar hobby-horse gait [22], rather than chorea. HDL3 represents a rare recessive, child-onset progressive disorder with chorea, dystonia, ataxia, spasticity, seizures and mutism [23]. Clinical manifestation of HDL4 or SCA17 often includes chorea, dystonia, parkinsonism, pyramidal signs, cognitive impairment, and psychiatric symptoms. Interestingly, pure parkinsonism is more common in patients with 41–45 repeats [24]. Since differentiation of SCA17 from HD is often challenging, the ocular motor findings can be utilized as a diagnostic marker, such as central positional nystagmus, more frequently observed in SCA17, or saccadic slowing in HD [25]. Recently, the concomitant presence of SCA17 with intermediate-length expansion (40–49) and heterozygous pathogenic mutation in the Stip1-homologous and U-Box containing protein 1 gene (*STUB1*) associated with SCA48 has also been described, with proposed digenic inheritance [26] or more likely the intermediate-length *TBP* expansion acting as a disease modifier of SCA48 [27]. Heterozygous *STUB1* variants were associated with a milder phenotype and reduced penetrance compared with the fully penetrant, complex cerebellar ataxia observed in cases cosegregating with intermediate-length *TBP* repeat expansions [28]. In SCA17, the age of onset and severity of the disease are negatively correlated with the length of the polyglutamine tract in *TBP*, although exceptions have been reported in the literature. Currently, the predominant molecular mechanism is thought to involve a toxic gain-of-function effect, through abnormal affinity of the mutant TBP protein for various proteins, disrupting their normal cellular functions [29].

### C9orf72-Related Neurodegenerative Disorders

The hexanucleotide repeat in the *C9orf72* gene was first linked to frontotemporal lobar degeneration (FTLD) and amyotrophic lateral sclerosis (ALS). However, it is now well-established as the most common HD phenocopy in the European population that should be investigated in first-line genetics testing [30]. Apart from chorea with predominant orofacial involvement, additional movement disorders such as tremor, parkinsonism or stimulus-sensitive upper limb myoclonus, can be the initial or only manifestation of the disease. Pathogenic expansions in *C9orf72* are known to drive disease through several interrelated mechanisms, including the formation of toxic RNA foci, the accumulation of dipeptide repeat (DPR) proteins generated by repeat-associated non-AUG (RAN) translation, impaired nucleocytoplasmic transport, and disturbances in protein homeostasis [31]. However, despite substantial progress in elucidating these core molecular mechanisms, the determinants of the marked phenotypic heterogeneity observed in *C9orf72*-associated disorders remain poorly understood, and the molecular factors that modulate clinical variability are still largely undefined [32]. Building on current mechanistic insights, emerging therapeutic strategies aim to target these pathogenic pathways through small molecules—such as G-quadruplex stabilizers, modulators of autophagy and proteasomal degradation, and inhibitors of RAN translation—as well as biological approaches, including antisense oligonucleotides and CRISPR-Cas–based gene editing, with the goal of directly modifying the underlying molecular defect [33].

### Neuroacanthocytosis Syndromes

Recent data significantly broaden the classical understanding of Chorea-acanthocytosis and McLeod syndrome. In chorea-acanthocytosis (ChAc), emerging evidence challenges the reliance on acanthocytosis and classical hyperkinetic presentation [34], as cases without detectable acanthocytes [35], and atypical phenotypes such as early-onset parkinsonism with dystonia, pyramidal signs, and myoclonus in the absence of prominent chorea further expand the clinical spectrum [36]. On the molecular level, altered sphingolipid and phospholipid composition [37] and variable lipid accumulation in affected brain tissue [36] suggest that disrupted lipid distribution is central to disease pathophysiology, positioning lipid homeostasis as a potential therapeutic target [38]. Beyond genetic confirmation, osmotic gradient ektacytometry of erythrocytes has emerged as a promising screening tool and potential biomarker [39].

In McLeod syndrome (MLS), a progressive supranuclear palsy-like phenotype has recently been described [40], as well as sleep apnea syndrome [41], broadening the neurological spectrum. Importantly, cases without typical Kell antigen abnormalities have been documented [42], challenging the traditional diagnostic clues. As recently shown, mitochondrial dysfunction may play a key role in the pathogenesis [43].

### Recent Advances in Brain Metal Accumulation Disorders Associated with Chorea

#### Wilson’s Disease

With its broad phenotypic presentation, Wilson’s Disease (WD) remains in the differential diagnosis for every choreatic patient, since therapeutic options are available. Moving towards the biomarkers era, blood-based markers such as active caspase-3, X-linked inhibitors of apoptosis protein (XIAP), pentraxin 3 (PTX3), neurofilament light chain (NfL), and pyrin domain-containing protein (NLRP3) inflammasome activation demonstrate strong potential for identifying neurological impairment. In hepatic manifestations, Macrophage Activation Marker sCD163 and Apoptosis Antigen 1 (APO-1) can be promising markers of liver dysfunction and fibrosis [44]. Interestingly, patients with loss-of-function (LOF) variants in *ATP7B* on chelators seem to have a worse prognosis and require close monitoring for signs of progressive liver disease [45]. After decades of minimal progress, advances in drug discovery have led to promising clinical trials of new therapeutic approaches, such as vector-based delivery of *ATP7B* variants, which have proven effective in mice and are currently moving forward to clinical applications, although the *ATP7B* gene seems too large for optimal adeno-associated virus vector delivery [46].

#### Primary Brain Calcification

So far, seven genes have been linked to Basal Ganglia Calcification syndrome. *SLC20A2*, *XPR1*, *PDGFB* and *PDGFRB* with autosomal dominant, or *MYORG*, *JAM2* and recently identified *NAA60* with autosomal recessive mode of inheritance [47]. Apart from chorea, parkinsonism or psychosis with manic features were also described [48]. To date, no disease-modifying therapy exists. However, a recent mouse study showed that splice-switching antisense oligonucleotides increased functional *SLC20A2* expression and, when administered intracerebroventricularly, reduced CSF phosphate levels and brain calcification in *SLC20A2* knock-in mice [49]. Disodium etidronate is also currently investigated in an ongoing CALCIFADE clinical trial [50].

#### Neuroferritinopathy

Heterozygous variants in the *FTL* gene cause hereditary neuroferritinopathy, a type of neurodegeneration with brain iron accumulation (NBIA), leading to adult-onset chorea or dystonia, with cognitive and behavioral issues [51]. Lately, mutations in the *FTH1* gene have also been linked to this disease in pediatric patients, with targeted antisense oligonucleotides’ knockdown of mutant *FTH1* transcript rescuing cellular phenotypes, suggesting a potential therapeutic strategy [52].

#### Benign Hereditary Chorea

Benign hereditary chorea (BHC) represents a very rare autosomal dominant disorder, where choreatic movements develop during infancy or adulthood. Chorea is usually very slowly progressive and without cognitive decline or other neurological features, though cases with dystonia, myoclonus or ataxia were also described. Currently, BHC is linked to mutations in *NKX2-1* and *ADCY5*. Recently, biallelic *HPCA* variants, originally associated with dystonia, have also been described in patients with BHC phenotype [53]. *NKX2-1*-related disorders range from BHC to choreoathetosis, congenital hypothyroidism, and neonatal respiratory distress syndrome (also known as brain-lung-thyroid (BLT) syndrome). Childhood-onset chorea, the hallmark feature of *NKX2-1*-related disorders, may or may not be associated with pulmonary disease or congenital hypothyroidism [54]. Dominant and, more rarely, also recessive [55], mutations in the *ADCY5* gene cause hyperkinetic movement disorder that can be either paroxysmal or permanent. The paroxysmal dyskinetic episodes can have multiple triggers and may occur at nighttime with perioral twitches and a good therapeutic effect after caffeine, which is often used as a diagnostic clue. Typically, symptoms are non-progressive, with onset in childhood and stabilization post-adolescence [56].

#### Chorea in Cerebellar Ataxia-Related Genes

Beyond SCA17, chorea, as the core phenotypic feature, is also described in several genes linked to cerebellar ataxia. For instance, chorea-athetosis is found in nearly all individuals with biallelic *ATM* mutations causing ataxia-telangiectasia (AT) and may be even the initial manifestation of the illness [57]. Chorea as the core feature has also been linked to Dentatorubral-pallidoluysian atrophy (DRPLA), often in combination with myoclonus epilepsy and dementia [58], SCA1, 2, 3, 8 and 12 [19], ataxia with oculomotor apraxia type 1, 2 and 4, Friedreich Ataxia [59] or Fragile X-associated tremor/ataxia syndrome (FXTAS) [60].

While these associations are well established, additional conditions linking ataxia and chorea are increasingly being recognized within the differential diagnostic spectrum of these disorders. These include the phenotypic spectrum of *RNF216*-related disease, which forms part of Gordon Holmes syndrome [61], characterized by the combination of ataxia and hypogonadotropic hypogonadism. In this context, chorea has recently been described as a frequent initial manifestation, particularly in individuals with disease onset after 30 years of age, and is often accompanied by cognitive decline [62].

Another important and recently delineated condition is Adult-onset neurodegeneration in nucleotide excision repair disorders (NERD), caused by pathogenic variants in *ERCC2*, *ERCC3*, *ERCC4*, *ERCC5*, and *XPA*. The core clinical features include ataxia and dementia, while chorea, spasticity, and peripheral neuropathy represent additional cardinal manifestations. Although previously considered rare, a recent landmark study demonstrated that these disorders accounted for 1% of previously unexplained ataxia cases, 3.5% of unexplained dementia cases, and 8.6% of unexplained cases presenting with combined ataxia and dementia in a cohort comprising 14,303 exome and genome datasets [63]. Importantly, associated cutaneous manifestations—most commonly photosensitivity, freckling, or skin neoplasms—may be subtle and easily overlooked [63–66].

Finally, chorea may also occur in certain acquired or hereditary immunodeficiency syndromes. X-linked hyper-IgM syndrome, caused by hemizygous variants in *CD40LG*, has been associated with neurological involvement in up to 15% of cases, with several reports describing adult-onset presentations characterized by cognitive decline, psychiatric disturbances, ataxia, and chorea [67–69].

#### Chorea in Epileptic Encephalopathy-Related Genes

Hyperkinetic movement disorders frequently overlap with epileptic or neurodevelopmental syndromes. Several genes implicated in early-onset epileptic encephalopathies are also responsible for isolated movement disorders, most frequently chorea and dystonia, such as *GNAO1* (Ohtatara syndrome), *FOXG1* (congenital Rett-like syndrome), *SCN8A* (paroxysmal kinesigenic dyskinesia and benign familial infantile seizures), *SYT1* (*ADCY5*-like phenotype with early onset, paroxysmal dyskinetic movement disorder worsening at night) [59], or *GABRB2 (*epileptic encephalopathy with developmental delay, choreoathetosis, dystonia and ataxia) [70]. Biallelic pathogenic loss-of-function variants in the *FRRS1L* gene are known to cause developmental and epileptic encephalopathy-37 (DEE37) with choreoathetosis or continuous spikes and waves during sleep (CSWS) [71]. Furthermore, chorea has been reported in association with pathogenic variants in *HSD17B10* gene, leading to refractory epilepsy and developmental delay [72].

#### Chorea in Dystonia-Related Genes

In addition to previously well-recognized dystonia-associated genes that may present with chorea such as *GNAO1*,* TOR1AIP2* gene, with a similar molecular effect as the well-established *TOR1A* gene, has been recently proposed as a candidate gene for dystonia-hemichorea/hemiballism syndrome [73]. Novel heterozygous *DRD2* variants were also described to cause juvenile-onset progressive chorea and dystonia phenotype [74, 75]. Interestingly, a mutation in the *ANO3* gene was found in a case of a childhood-onset chorea-dominant phenotype that later evolved into a dystonia-dominant phenotype [76].

#### Chorea in Neurodevelopmental Disorders-Related Genes

Several other genes have recently been implicated in disorders in which chorea represents a predominant feature, underscoring the expanding phenotypic overlap among chorea, ataxia, epilepsy and neurodevelopmental syndromes. A novel biallelic variant in the *TSFM* gene, previously associated with lethal complex infantile-onset disease, was currently linked to early-onset generalized chorea with myoclonus, dystonia, cerebellar symptoms, and slowly progressive optic atrophy, accompanied by symmetric nodular hyperintensities in the subthalamic nuclei on brain MRI [77]. Notably, cases without basal ganglia lesions were also reported [78]. An emerging role of cyclic nucleotide phosphodiesterase (PDE) enzymes in the pathophysiology of movement disorders is also being increasingly recognized. Pathogenic variants in *PDE2A*, *PDE8B*, and *PDE10A* are responsible for rare forms of developmental delay, parkinsonism and chorea [79, 80]. The phenotypic spectrum of *PDE10A-*childhood-onset chorea, which could also involve dystonia [81], was recently expanded by peculiar foot deformities and pronounced social phobia, with brain MRI missing the characteristic symmetrical bilateral striatal lesions [82]. A deleterious biallelic mutation in the *GPR88* gene was also identified in familial cases characterized by developmental delay and choreatic features [83]. Recently, a bi-allelic loss-of-function mutation in the *ACBD6* gene was found in cases with developmental delay and a broad spectrum of both hypo- and hyperkinetic movement disorders, including chorea-like jerky movement and vocal tics like those in ChAc [84]. *RHOBTB2*-related disorder (RRD) is another example that should be considered in patients with neurodevelopmental delay in combination with epileptic encephalopathy or movement disorder such as chorea or severe paroxysmal dyskinesia [85]. Additional features of RRD include intellectual disability, microcephaly and brain malformation, behavioral dysregulation and dysmorphisms [86]. Chorea was even observed in the *NOTCH3* mutation carrier with a brain MRI scan presenting typical cerebral microvascular ischemic change affecting the lentiform nucleus, thalamus and brainstem [87].

### Strategies for Genetic Testing in Chorea-Related Disorders

Given the expanding genetic landscape of chorea, a phenotype-driven approach has become essential in clinical practice. In adult-onset progressive chorea, the first-line testing should exclude *HTT* CAG repeat expansion. If negative, further evaluation should exclude HDL, DRPLA, as well as *C9orf72* repeat expansion, particularly in European population. Importantly, repeat expansions may not be reliably detected by standard short-read sequencing methods and often require repeat-primed PCR or long-read approaches. In childhood- or early-onset chorea, WD should not be missed in first-line testing, Systematic features should further guide the possible diagnosis of neuroacanthocytosis syndromes or benign hereditary chorea. With the increasing number of newly-identified genes associated with chorea, whole-exome (WES) and increasingly whole-genome sequencing (WGS), particularly for intronic or structural mutations, are becoming standard diagnostic tools. Focused gene panels may miss the condition previously associated with pure ataxia, dystonia or neurodevelopmental phenotypes, underscoring the need for broader diagnostic strategies in complex cases. However, a recent case report of *NKX2-1* mutation demonstrated that even new sequencing technologies missed discrete mobile element insertion leading to clinical presentation of BLT syndrome [88].

## Conclusions

Chorea is increasingly recognized across a broad and expanding spectrum of genetic disorders. Advances in genomic technologies and the establishment of large patient cohorts led to the identification of several novel genetic conditions, including those associated with e.g. T*OR1AIP2*, *FTH1*, *NAA60* or *ACBD6*,* as well as newly delineated* phenotypes such as NERD-ND. Furthermore, improved recognition and systemic reporting of movement disorders in complex conditions - particularly those typically linked to neurodevelopmental disorder (cerebral palsy like syndromes), epileptic encephalopathies, and immunodeficiencies - have further expanded the list of diseases linked to chorea. As the differential diagnosis of chorea disorders becomes more complex, the development of a revised classification system would be highly valuable. A two axis framework, analogous to existing classification systems for tremor [1] and dystonia [2] - including clinical characteristics and etiology - could provide a structured approach to the diagnostic process and clinical management. In parallel, several major advancements in the understanding of chorea pathophysiology have emerged over recent years. Notably, increasing insight into the role of somatic instability in Huntington’s disease (HD) holds significant therapeutic implications, not only for HD but also for other repeat expansion disorders. Moreover, improved understanding of converging molecular pathways and advancements in gene therapies may pave the way for a more personalized therapeutic approach for genetic choreas in the upcoming years [89].

## Data Availability

No datasets were generated or analyzed during the current study.

## Key References

- Handsaker RE, Kashin S, Reed NM, Tan S, Lee WS, McDonald TM, Morris K, Kamitaki N, Mullally CD, Morakabati NR, Goldman M, Lind G, Kohli R, Lawton E, Hogan M, Ichihara K, Berretta S, McCarroll SA. Long somatic DNA-repeat expansion drives neurodegeneration in Huntington's disease. Cell. 2025 Feb 6;188(3):623-639.e19. 10.1016/j.cell.2024.11.038.
  - This manuscript outlines the role of somatic instability in the pathophysiology of HD and proposes a novel model ELongATE which outlines the biological effects of expanding CAG repeats in striatal projection neurons. These results may have major implications not only in HD, but also in other repeat expansion disorders.
- Cordts I, Önder D, Traschütz A, Kobeleva X, Karin I, Minnerop M, et al. Adult-Onset Neurodegeneration in Nucleotide Excision Repair Disorders (NERDND ): Time to Move Beyond the Skin. Mov Disord. 2022 Aug;37(8):1707-1718. 10.1002/mds.29071.
  - The authors outline a novel diagnostic entity Adult-Onset Neurodegeneration in Nucleotide Excision Repair Disorders (NERD-ND), due to mutations linked to Xeroderma pigmentosum genetic spectrum, nevertheless with dominant neurological symptoms, typically including dementia, ataxia and chorea, and less prominent dermatological findings.
- Kaiyrzhanov R, Rad A, Lin SJ, Bertoli-Avella A, Kallemeijn WW, Godwin A, et al. Bi-allelic ACBD6 variants lead to a neurodevelopmental syndrome with progressive and complex movement disorders. Brain. 2024 Apr 4;147(4):1436-1456. 10.1093/brain/awad380.
  - This manuscript outlines a large cohort of cases with novel neurodevelopmental syndrome with progressive and complex movement disorders including chorea due to bi-allelic ABCD6 variants. The authors provide detailed phenotypic and functional data.
- Kafantari E, Hernandez VJ, Necpál J, Leonidou M, Baureder R, Hedberg-Oldfors C, et al. TOR1AIP2 as a candidate gene for dystonia-hemichorea/hemiballism. Parkinsonism Relat Disord. 2025 May;134:107781. 10.1016/j.parkreldis.2025.107781.
  - This manuscript proposes a novel candidate gene for dystonia-hemichorea disorder due to hemizygous TOR1AIP2. Two families and functional data are described.
- Shieh JT, Tintos-Hernandez JA, Murali CN, Penon-Portmann M, Flores-Mendez M, Santana A, et al. Heterozygous nonsense variants in the ferritin heavy-chain gene FTH1 cause a neuroferritinopathy. HGG Adv. 2023 Oct 12;4(4):100236. 10.1016/j.xhgg.2023.100236.
  - The authors describe mutations in FTH1 encoding the heavy unit of ferritin as a novel human genetic condition causing neuroferritinopathy in pediatric patients. Detailed clinical, imaging and functional data are provided.
